# Characterisation and modelling of continuous electrospun poly(ɛ- caprolactone) filaments for biological tissue repair

**DOI:** 10.1016/j.jmbbm.2024.106810

**Published:** 2025-01

**Authors:** Thales Zanetti Ferreira, Zhouzhou Pan, Pierre-Alexis Mouthuy, Laurence Brassart

**Affiliations:** a Department of Engineering Science, University of Oxford, Oxford OX1 3PJ, United Kingdom; b Botnar Institute of Musculoskeletal Sciences, Nuffield Department of Orthopedics, Rheumatology and Muscoskeletal Sciences, University of Oxford, Oxford, OX3 7LD, United Kingdom

**Keywords:** Electrospinning, Biomedical fibres, PCL, Viscoelasticity, Viscoplasticity, Mechanical characterisation

## Abstract

This study investigates the mechanical behaviour of poly(ɛ-caprolactone) (PCL) continuous filaments produced by a novel electrospinning (ES) method. These filaments can be processed into woven or braided structures, showing great promises as scaffolds for ligament and tendon repair. Mechanical characterisation of the filaments using DMA and uniaxial tensile tests shows that the filament response is viscoelastic–viscoplastic. Filaments tested using bollard grips present an initially linear elastic response, followed by plastic yielding with two-stage hardening. The filaments are highly stretchable, reaching more than 1000% strain. The different deformation stages are correlated to the evolution of the micro-fibre network observed using SEM, involving the untangling, alignment and stretching of the fibres. A large deformation viscoelastic–viscoplastic model is proposed, which successfully captures the mechanical response of the filaments under non-monotonic loading conditions. Our study also highlights the sensitivity of the measured mechanical response to the type of mechanical grips, namely bollard or screw-side grips.

## Highlights

• Continuous PCL filaments promising for ligament and tendon repair.
• Thermo-mechanical characterisation (DSC, DMA) of pre-stretched filaments.
• Viscoelastic, viscoplastic response well captured by constitutive model.
• Deformation stages correlated to evolutions at the micro-fibre network level.
• Measured stress–strain curves sensitive to grip type (bollard or screw-side).

## Graphical abstract

**Figure:**
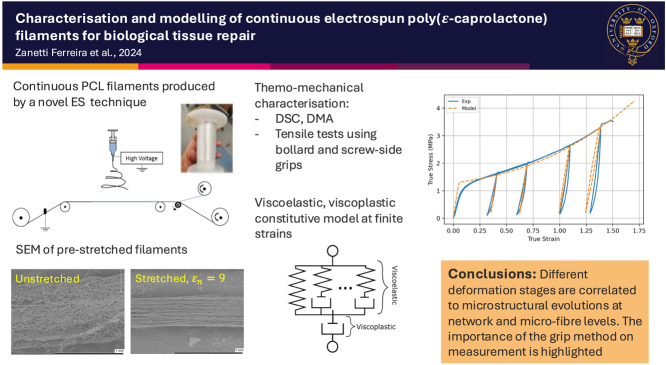

## 1 Introduction

Electrospinning (ES) is a simple yet powerful method for producing ultra-lightweight non-woven textiles made of micro- to nano-sized fibres (Ratner et al., 2004, Amoroso et al., 2011, Bosworth, 2014). This technique utilises electrostatic forces to draw fibres from a viscoelastic polymer solution. The drawn fibres are continuously deposited onto a collecting device to produce an interconnected non-woven mesh (Fang et al., 2008). Due to their highly porous structure, high surface-to-volume ratio, and tunable architecture, ES materials have been used in a broad range of applications, including biomedical, pharmaceutical, packaging, and textile applications (Zhao et al., 2020, Nangare et al., 2020, Liu et al., 2021, Fang et al., 2008, Liu et al., 2020). ES meshes are particularly attractive for tissue engineering applications, where they can be designed to mimic the extracellular matrix architecture, promoting tissue growth (Liu et al., 2020, Mouthuy et al., 2015, Sensini and Cristofolini, 2018).

In the search for robust and scalable ES methods, a novel automated technique was recently introduced by Mouthuy et al. (2015) to produce continuous ES filaments by depositing the fibres onto a continuous guiding wire. This technique differs from traditional ES setups, which collect fibres onto the surface of plates, bars, and dikcs (Teo and Ramakrishna, 2006). Compared to planar constructs, the advantage of continuous ES filaments is that they can be further processed into woven, knitted, or braided structures, which can be used to create a variety of scaffolds. For example, multifilament yarns made of twisted or woven ES filaments have shown great promise for ligament and tendon repair (Mouthuy et al., 2015, Savić et al., 2021). However, the mechanical behaviour of such ES filaments has not been fully characterised and understood.

The objective of this work is to characterise the thermo-mechanical behaviour of poly(ɛ-caprolactone) (PCL) ES filaments produced following the technique developed by Mouthuy et al. (2015). Previous studies investigated the mechanical response of polydioxanone ES filaments in the context of suture applications (Lach et al., 2019, Abhari et al., 2017, Abhari et al., 2018a, Abhari et al., 2018b). Here, PCL was selected due to its wide use in tissue engineering, exhibiting a slow degradation rate and minimal inflammatory response (Bartnikowski et al., 2019, Malikmammadov et al., 2018). Thermo-mechanical characterisation tests considered in this study include Differential Scanning Calorimetry (DSC), Dynamic Mechanical Analysis (DMA), as well as quasi-static uniaxial tension tests. Our results show that the filament response is rate-dependent and involves plastic yielding followed by re-hardening at large deformations. The different stages of the deformation are correlated to the evolution of the microscopic fibrous network, observed using Scanning Electron Microscopy (SEM). The sensitivity of the measured mechanical response to the type of mechanical grips used is highlighted. We further develop a viscoelastic–viscoplastic constitutive model to phenomenologically capture the mechanical response.

## 2 Experimental methods

### 2.1 Polymer solution preparation

Electrospinning solutions were prepared by dissolving PCL with molecular weight $M_{w}=$ 167 kDa (Ashland Specialities, Ireland) into 1,1,1,3,3,3-hexafluoroisopropanol (HFIP) (Apollo Scientific Ltd, UK). The weight-to-volume ratio of PCL to HFIP was 10%. Solutions were agitated at room temperature at a speed of 21 rpm using a roller mixer (Stuart SRT9D, UK) for at least 24 h to ensure complete dissolution of the polymer.

### 2.2 Electrospinning

**Fig. 1:**
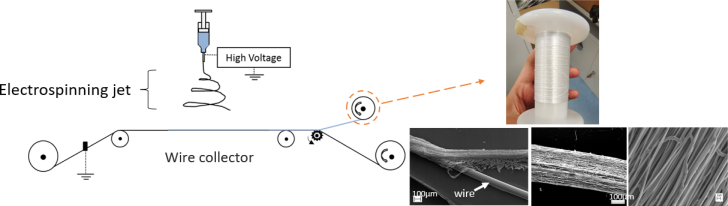
Schematic of the novel electrospinning collection technique. The method consists of electrospinning PCL fibres onto a stainless steel wire progressing at a speed of 0.4 mm s^−1^. The electrospun mesh is separated from the wire and collected in the form of a continuous filament.

ES filaments were produced by depositing a continuous fibre on a thin guided wire, creating a dense and narrow mesh that can be detached as a long continuous thread, as shown in Fig. 1. A detailed description of the production technique can be found in Mouthuy et al. (2015). Electrospinning was performed with a single nozzle setup, a continuous wire (100μm diameter) (Goodfellow, UK), an SL30P30/230 high voltage power supply (30 kV)(Spellman, UK) and a syringe pump (World Precision Instruments Limited, US). The metallic wire was cleaned with 70% ethanol prior to setup. The process was conducted inside a glove box under constant airflow to prevent organic vapour from interacting with the process. Environmental factors were controlled with the room temperature set to 22±3°C and humidity to 40±3%. The polymer solution was fed at a rate of 1 ml h^−1^. The distance between the nozzle tip and wire was 20 cm and the applied voltage ranged between 7 kV and 10.5 kV. The wire displaced perpendicular to the nozzle tip at a speed of 0.4 mm s^−1^. The resultant product is a metallic wire coated in a continuous ES mesh. Following the exit of the wire from the glovebox, the ES filament was detached from the wire and automatically collected on a rotating spool. The filament spool was stored in a desiccator until required for characterisation testing.

### 2.3 Thermal properties

Differential scanning calorimetry (DSC) was used to investigate the thermal properties of ES PCL filaments. Experimental tests were performed with a DSC Q2000 differential scanning calorimeter (TA Instruments, US) in a nitrogen atmosphere with a flow rate of 50 mL min^−1^ and standard aluminium cups with lid. Prior to testing, samples were stretched at an extension rate of 75 mm min^−1^ to different nominal strains (${ɛ}_{n}=0$, 1, 2, 4, 7, and 9) using bollard grips, (see Section 2.4) to examine whether stretching induced an increase in crystallinity. Three samples were tested for each strain. Each sample was first cooled to reach a thermal equilibrium of −90 °C, then heated at 10 °C min^−1^ up to 80 °C. Samples were then cooled at 10 °C min^−1^ down to −30 °C. The heat flow per unit mass (W g^−1^) was recorded to identify any phase transformations. DSC thermographs were analysed using TA Universal Analysis software.

### 2.4 Mechanical testing

**Fig. 2:**
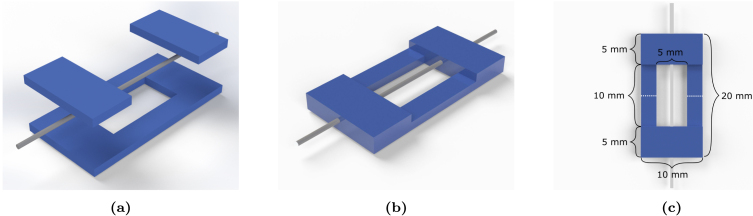
(a) Electrospun filaments were cut to a standardised size of 25 mm and placed on top of a pre-cut window-style paper cardboard frame. Filaments were glued onto the frame. (b) An additional layer of cardboard paper was glued on top of the filament. (c) The frame was mounted onto testing apparatus with a 10 mm gauge length and the cardboard frame was cut along the white discontinuous lines.

The small-strain viscoelastic properties of filaments were characterised via dynamic mechanical analysis (DMA). Uniaxial tension tests were performed using a Q800 dynamic mechanical analyser (TA Instruments, US) using a thin-film clamp at room temperature (27 ± 2 °C) and environmental humidity conditions. Samples were prepared by glueing filaments onto a cardboard window frame to increase the clamping surface area, as illustrated in Fig. 2. Temperature sweep tests ranging between −92 °C and 46 °C were conducted with six frequencies ranging between 0.1 Hz and 10 Hz and a 0.1% strain. The storage modulus ($E^{\prime }$), loss modulus ($E^{\prime \prime }$), and loss tangent (tanδ) were recorded.

The mechanical response of ES PCL filaments at large strains was characterised using an Instron 5582 electromechanical tensile tester (Instron, UK) with a 100 N load cell. Tests were performed with two different grip methods outlined in ISO 2307:2019 (ISO, 2019).

**Fig. 3:**
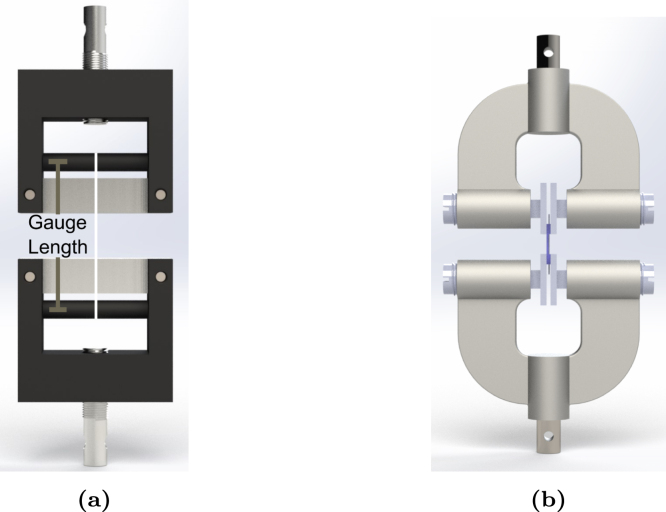
Illustration of (a) bollard grips and (b) screw-side tensile grips.

(i) **Bollard grips:** Filaments were cut to a standardised length of 120 mm as this was the minimum length required to wrap the filaments around the grips. Filaments were first wrapped around the top bollard and clamped. Wrapping around the lower component was carefully conducted to ensure vertical alignment and prevent any torsion of the filament. A 45 mm gauge length was defined by the two contact points at which the filament tangentially contact the grips, as illustrated in Fig. 3(a). Mounting the filaments introduced some pre-load in the range 0.05–0.1 N.
(ii) **Screw-side grips:** Samples tested with screw-driven tensile grips were prepared with the same cardboard methodology as outlined in Fig. 2. The gauge length was 10 mm. The cardboard frame was mounted onto the grips and tightened using the screws, as illustrated in Fig. 3(b).

Uniaxial tensile tests were conducted at room temperature and environmental humidity conditions. Filaments were tested at extension rates of 5, 75, and 150 mm min^−1^ until failure. The nominal strain was defined as ${ɛ}_{n}=\frac{L-L_{0}}{L_{0}}=\lambda -1$, with $L_{0}$ and L the initial and deformed gauge lengths, respectively, and λ is the filament stretch. The nominal stress was obtained by dividing the force by the initial cross-section area, calculated from the average apparent diameter of the filaments measured by SEM, see Section 2.5, and assuming that the filament cross-section is circular. For each extension rate, at least six samples were tested, and the force (N), elongation (mm) were recorded as a function of time. The apparent elastic modulus was calculated by fitting a linear regression in the linear elastic regime of each sample. The yield stress was then estimated as the intersection between the regression model and experimental data with a 0.2% strain offset. Loading/unloading tests at 75 mm min^−1^ were performed to examine the filament plastic behaviour. The relaxation behaviour of the filaments was investigated by loading the filament at 75 mm min^−1^ up to a given force value. The corresponding displacement was then held constant and the force measured during relaxation. Cyclic loading tests were conducted on samples pre-stretched by eight times their original length. Tests were performed at 75 mm min^−1^ and for 10 cycles.

Statistical analysis was performed using OriginPro (OriginLab Corp., US). Unpaired t-tests were performed using one-way ANOVA and Tukey methods to examine the statistical differences between two independent groups of unequal sample size. Results were considered significant for *p* ≤ 0.05.

### 2.5 Microstructural characterisation

The microstructure of pre-stretched filaments was characterised using a TM3030Plus tabletop scanning electron microscope (Hitashi, Japan). Independent filaments were first stretched to specific strains using an electromechanical tensile testing with a 75 mm min^−1^ extension rate. Filaments were then unmounted from the machine and given time to fully relax (at least 30 min). The middle portion of each stretched filament was then cut in several shorter sub-filaments using scissors. The sub-filaments were mounted onto an aluminium stub using a carbon adhesive disk. The SEM was operated under variable pressure with a 15 kV acceleration voltage and 4.4–4.8 mm working distance. Micrographs were taken at magnifications between ×100 and ×5000. The filament and fibre apparent diameters were measured using the ImageJ software. All average diameter values reported in the paper were calculated based on micrographs corresponding to three different sub-filaments, and at least 10 diameters were measured in each micrograph. Surface porosity was estimated based on the area fraction of the dark region relative to the white fibrous region in contrasted images in ImageJ. The reported porosity values presented in the paper were obtained based on a single micrograph.

Alternatively, the continuous evolution of the filament microstructure during deformation was characterised through *in-situ* tensile testing. Samples were tested with a Deben Microtest tensile stage (Judges Scientific, UK) with a 20 N load cell. The stage was designed to fit into an Evo LS15 Environmental SEM (Carl Zeiss, Germany) for *in-situ* microscopy testing. Specimens were prepared using the cardboard method outlined in Fig. 2 and required no metallic coating as the SEM was operated under environmental variable pressure at 15 kV acceleration voltage and 14 mm working distance. Samples were stretched at a deformation rate of 5 mm min^−1^ and held at constant strain while images were taken.

## 3 Experimental results

### 3.1 Thermal properties

**Fig. 4:**
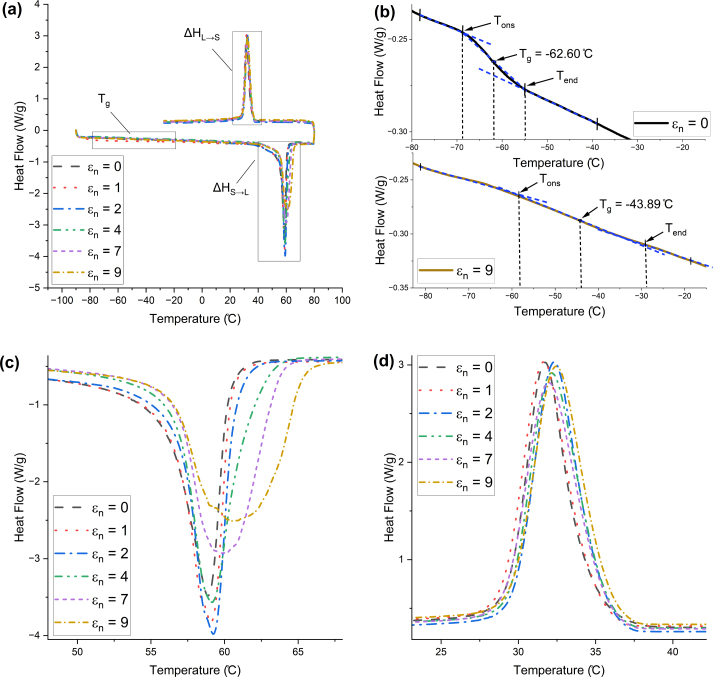
(a) DSC thermographs for electrospun PCL filaments at different level of pre-strain (${ɛ}_{n}$ = 0, 1, 2, 4, 7, and 9). Exothermic reaction upwards. (b) Zoom on the glass transition region for unstrained filaments (top) and filaments with a prestrain of 9 (bottom). (c) Zoom on the endothermic (i.e. melting) peaks during heating. (d) Zoom on the exothermic (i.e. recrystallisation) peaks during cooling.

Fig. 4(a) shows DSC thermographs of the average response of ES filaments stretched to six different nominal strains (${ɛ}_{n}$ = 0, 1, 2, 4, 7, and 9). Each curve corresponds to the average of three filament responses. Thermographs all show the same response overall, although two subtle differences arise from stretching the filaments. Firstly, the heat flow curve in the glass transition region presents an ‘S’ shape for the un-stretched filaments, whereas it gradually becomes linear as the level of pre-strain increases (Fig. 4(b)). Secondly, the endothermic heat flow peak amplitude gradually reduces and its spread increases with increasing strain (Fig. 4(c)). However, the exothermic (i.e. recrystallisation) peak appears largely independent of the level of pre-strain (Fig. 4(d)).

The glass transition temperature $T_{g}$ was determined using a conventional tangent construct method (Menczel and Prime, 2009), illustrated in Fig. 4(b). First, two tangents to the heat flow curve were drawn, passing through temperature points respectively before and after the glass transition. A third construct was drawn tangent to the heat flow curve in the transition region. Intersection points between the tangents defined the extrapolated onset and endset temperatures, $T_{ons}$ and $T_{end}$, and the glass transition temperature was obtained as the midpoint between these two temperatures (“half-width” method). The melting temperature $T_{m}$ was identified as the minimum value of the endothermic peak. The enthalpy of fusion from solid to liquid ($\Delta H_{S\to L}$) and recrystallisation enthalpy ($\Delta H_{L\to S}$) were determined by from areas under the respective peaks. The results are summarised in Table 1. Un-stretched samples present a glass transition temperature of −62 ± 2 °C and a melting temperature of 58 ± 2 °C. These results are consistent with bulk material properties for PCL reported in the literature: $T_{g}\approx -60\;{}^{\circ}C$ and $T_{m}\approx 60\;{}^{\circ}C$ (Bartnikowski et al., 2019). This indicates that electrospinning does not affect the molecular structure of the drawn fibres. The glass transition and melting temperatures increase with the degree of pre-strain.

The degree of crystallinity %K of the ES samples was estimated using the following formula:

(1) $$\text{\%}K=\frac{\Delta H}{\Delta H_{0}}$$

where ΔH is the experimentally-measured melting enthalpy ($\Delta H_{S\to L}$ or $\Delta H_{L\to S}$), and $\Delta H_{0}$ is the reference value for 100% crystalline material, reported as 139.5 J g^−1^ (Pitt et al., 1981, Lam et al., 2008). Un-stretched samples showed a 61 ± 1% crystallinity, which progressively increased up to 69% at a strain of 9, indicating some degree of strain-induced crystallisation. This is consistent with the increase in $T_{g}$ as the pre-strain increases, since aligned molecules in the crystalline phase have reduced mobility. On the other hand, the degree of crystallinity reached upon cooling the material from the melt was about 47 ± 2%, independent of the level of pre-strain. Comparing the degrees of crystallinity in an ES filament and a recrystallised filament at zero strain, the electrospinning process contributes to an approximate 13% increase in crystallinity.

**Table 1:** The glass transition temperature ($T_{g}$), melting temperature ($T_{m}$), enthalpy of fusion from solid to liquid ($\Delta H_{S\to L}$), the recrystallisation enthalpy ($\Delta H_{L\to S}$), and degree of crystallinity calculated from $\Delta H_{S\to L}$ and $\Delta H_{L\to S}$, respectively, for pre-stretched filaments.

${ɛ}_{n}$	$T_{g}$ (°C)	$T_{m}$ (°C)	$\Delta H_{S\to L}$ (J g${}^{-1}$)	$\Delta H_{L\to S}$ (J g${}^{-1}$)	% Crystallinity $\Delta H_{S\to L}$	% Crystallinity $\Delta H_{L\to S}$
0	−62.60	58.88	85.96	67.61	61.62%	48.47%
1	−63.42	59.08	88.24	65.18	63.25%	46.72%
2	−62.58	59.24	90.62	66.14	64.96%	47.41%
4	−58.88	59.14	91.27	65.23	65.43%	46.76%
7	−45.81	60.11	92.11	65.69	66.03%	47.09%
9	−43.89	60.54	97.03	67.67	69.56%	48.51%

### 3.2 Viscoelastic properties

**Fig. 5:**
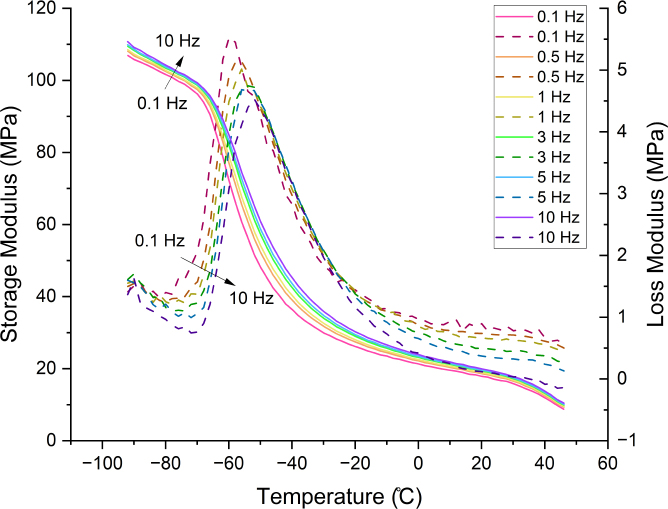
Storage modulus ($E^{\prime }$, solid lines) and loss modulus ($E^{\prime \prime }$, dashed lines) of un-stretched electrospun PCL filaments measured by DMA in temperature sweeps at 0.1, 0.5, 1, 3, 5, and 10 Hz.

The storage modulus, loss modulus, and loss tangent measured by DMA in temperature sweeps at varying frequencies are shown in Fig. 5. The glass transition temperature at each loading frequency was estimated in three different ways: (i) from the drop in storage modulus, (ii) from the loss modulus peak, and (iii) from the loss tangent peak, and values are reported in Table 2. Regardless of the method, the glass transition temperature increases with the loading frequency. The frequency dependence of the glass transition temperature results from the kinetic and relaxation character of the glass transition (Perez, 2018). We also find that the glass transition temperature of un-stretched filament measured from DSC is intermediate between the values obtained from the storage and loss moduli curves, whereas the glass transition temperature obtained from the loss tangent peak is systematically larger than the corresponding value measured from DSC. Variations in measured values of $T_{g}$ using the three techniques have been attributed to the broad temperature range of the transition region and the fact that different definitions reflect different aspects of the transition (Hagen et al., 1994).

**Fig. 6:**
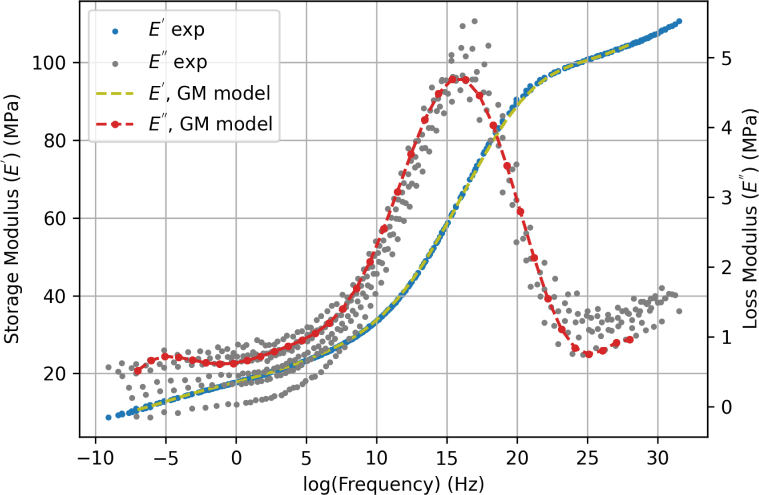
Master curves for the storage ($E^{\prime }$) and loss ($E^{\prime \prime }$) modulus as a function of the loading frequency. Experimental master curves are fitted using the Generalised Maxwell (GM) model.

We used the Time-Temperature Superposition (TTS) principle (Ward and Sweeney, 2012) to obtain the frequency dependence of $E^{\prime }$ and $E^{\prime \prime }$ from temperature sweeps reported in Fig. 5. For each temperature, data for the storage modulus were first plotted as a function of the logarithm of the frequency, and then shifted to produce a smooth master curve at the reference temperature 26.1 °C. The same shift factors were then used to obtain the corresponding master curve for the loss modulus. Experimental master curves are shown in Fig. 6. We then fitted the experimental master curves for the storage and loss moduli using the generalised Maxwell model:

(2) $$E^{\prime }=E_{\infty }+\overset{N}{\underset{i=1}{\sum }}E^{\left(i\right)}\frac{\omega^{2}{\left(\tau^{\left(i\right)}\right)}^{2}}{1+\omega^{2}{\left(\tau^{\left(i\right)}\right)}^{2}},$$

(3) $$E^{\prime \prime }=\overset{N}{\underset{i=1}{\sum }}E^{\left(i\right)}\frac{\omega \tau^{\left(i\right)}}{1+\omega^{2}{\left(\tau^{\left(i\right)}\right)}^{2}},$$

where ω is the angular frequency, $E^{\left(i\right)}$ and $\tau^{\left(i\right)}$ are the elastic constant and relaxation time for each Maxwell element, and $E_{\infty }$ is the relaxed modulus. We found that N=37 Maxwell elements were needed to correctly fit the master curves, see Fig. 6. Fitted parameters are reported in Table A.6. The large number of relaxation times needed is explained by the very broad range of frequencies resulting from application of the TTS principle, spanning 4 decades.

**Table 2:** The glass transition temperature ($T_{g}$) values determined from DMA measurements of the storage and loss modulus.

Frequency (Hz)	$T_{g}$ (°C) determined from $E^{\prime }$	$T_{g}$ (°C) determined from $E^{{}^{\prime \prime }}$	$T_{g}$ (°C) determined from tanδ
0.1	−67.09	−59.93	−51.96
0.5	−66.37	−55.92	−47.94
1	−65.50	−55.93	−47.94
3	−65.08	−53.92	−45.92
5	−64.98	−55.94	−47.94
10	−64.21	−51.93	−45.93

### 3.3 Mechanical properties

**Fig. 7:**
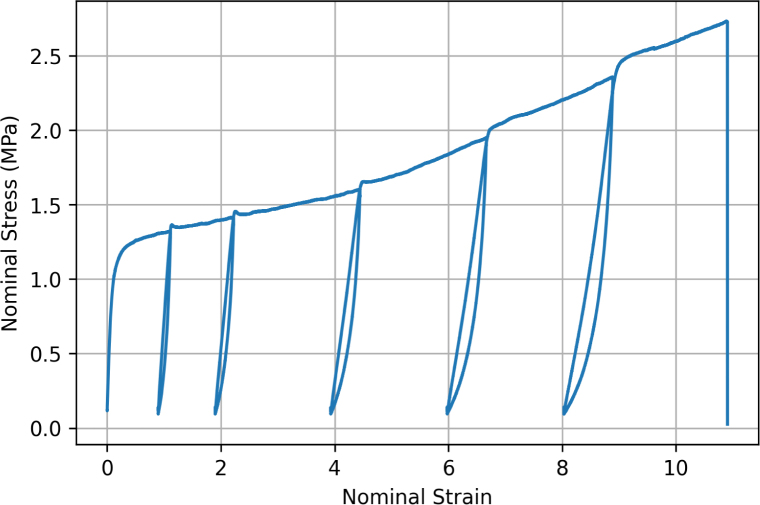
Tensile response of a filament tested using bollard grips at 75 mm min^−1^. The unloading/reloading sequences were performed at nominal strains of 1, 2, 4.5, 6.5, and 9.

The characteristic stress–strain response of a representative ES PCL filament, tested using bollard grips under loading/unloading conditions at 75 mm min^−1^ is illustrated in Fig. 7. The stress–strain profile can be decomposed into (i) a linear elastic region and (ii) a plastic region with two-stage hardening, until failure which occurred randomly in the gauge region. The linear region is characterised by an apparent elastic modulus of 9.1 ± 1 MPa (here and for other mechanical properties, the average value and standard deviation were calculated based on the response of at least 7 filaments). The filament yields at around 0.9±0.2MPa. Subsequently, the filament plastically deforms until reaching a nominal strain of 11±1, with maximum nominal stress reaching 2.5±0.2MPa.

To obtain the loading/unloading response, a single filament was tested in uniaxial tension up to a pre-determined value of nominal strain (${ɛ}_{n}$ = 1, 2, 4.5, 6.5, and 9), before unloading and reloading. The reversal stress was set to 0.1 MPa because below this value the sample would lose tension, causing the sample to detach from the grips and the software would recognise it as a fractured sample. Loading/unloading results shown in Fig. 7 confirm that filaments are subjected to plastic deformation, which increases with the applied strain. Furthermore, each deformation cycle displayed a small hysteresis loop, which progressively increases with the applied strain, indicating that the mechanical response of the filaments is viscoelastic.

**Fig. 8:**
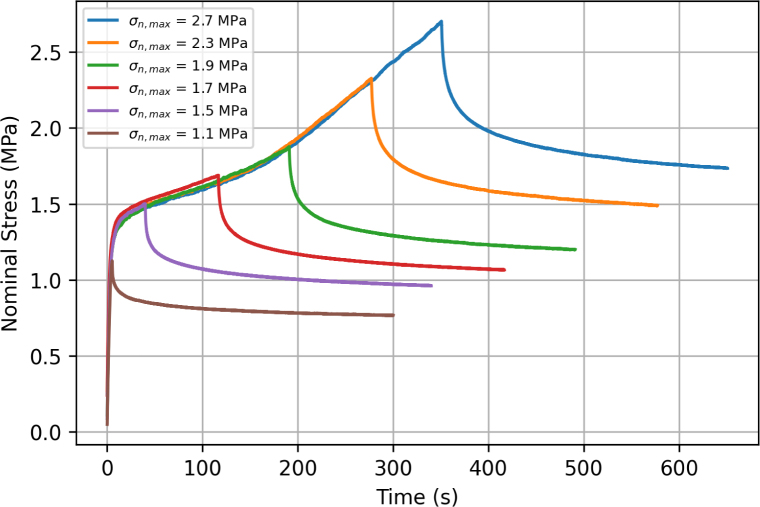
Stress relaxation response of filaments stretched using bollard grips at 75 mm min^−1^, up to maximum stresses 1.1, 1.5, 1.7, 1.9, 2.3, and 2.7 MPa, and let to relax under constant displacement for five minutes.

Fig. 8 shows the stress relaxation response of six independent samples extended at 75 mm min^−1^ up to a force of 0.6, 0.8, 0.9, 1, 1.2, and 1.4 N, respectively, corresponding to a maximum nominal stress $\sigma_{n,max}$ = 1.1, 1.5, 1.7, 1.9, 2.3 and 2.7 MPa, with displacement kept constant afterwards for five minutes. Results confirm that filaments are viscous and exhibit stress relaxation. The relaxation response can be decomposed into an instantaneous relaxation reaction followed by a long-term plateau. To evaluate differences in relaxation response with deformation, the percentage change in stress during relaxation was recorded, based on the peak stress and the last recorded stress values. The stress drop ranged from 31.88% for $\sigma_{n,max}$ = 1.1 MPa to 35.79% for $\sigma_{n,max}$ = 2.7 MPa.

### 3.4 Rate sensitivity and experimental variability

**Fig. 9:**
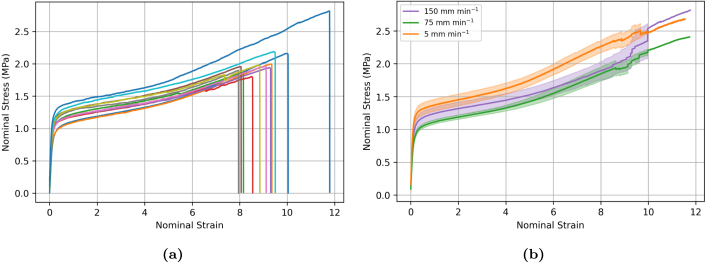
(a) Illustration of experimental variability in the stress–strain response of electrospun filaments tested at 150 mm min^−1^ using bollard grips. (b) Average response for at least seven filaments tested at extension rates of 5, 75, and 150 mm min^−1^. Surrounding shadowing represents the standard deviation.

Fig. 9(a) illustrates the experimental variability in the stress–strain response measured using bollard grips. The figure shows the stress–strain response of seven independent samples tested at 150 mm min^−1^. Although filaments demonstrate a similar overall response, they exhibit different yield, ultimate stress and strain values. A similar variability is observed at other loading rates (not shown). The experimental variability may be attributed to several factors. Firstly, the manipulation of filaments during mounting to achieve precise positioning and alignment may lead to pre-stretching and pre-stresses that may impact the recorded stress–strain values. Secondly, all stress–strain curves use the same averaged reference diameter for nominal stress calculation. Lastly, the mechanisms through which fibres are recruited and activated with the load may vary from sample to sample due to its random nature.

**Fig. 10:**
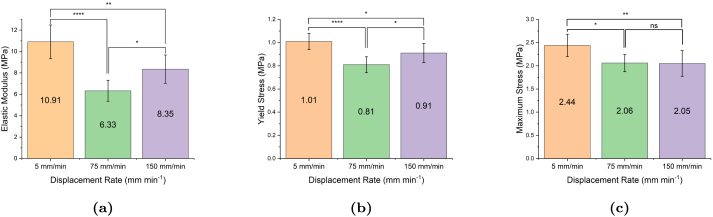
Summary statistics on the effect of displacement rate on the (a) elastic modulus, (b) yield stress ($\sigma_{y}$), and (c) maximum tensile stress ($\sigma_{max}$). Error bars represent standard deviation. ns = not significant, *p<0.05, **p<0.01, ***p<0.001, ****p<0.0001.

Fig. 9(b) shows the averaged uniaxial response for at least seven filaments at extension rates of 5, 75, and 150 mm min^−1^. The shadowed region represents the standard deviation at each strain. Corresponding values of apparent elastic modulus, yield stress and maximum tensile stress are reported in Fig. 10. Although results suggest that the response is rate-dependent, the observed rate-dependency does not follow the expected trend that samples tested at a higher displacement rate experience higher stresses. In particular, samples tested at displacement rates of 5 mm min^−1^ were stiffer and stronger on average than samples tested at the faster rates of 75 and 150 mm min^−1^. Statistical analysis results shown in Fig. 10 show that the measured differences between properties at different displacement rates are statistically significant.

**Fig. 11:**
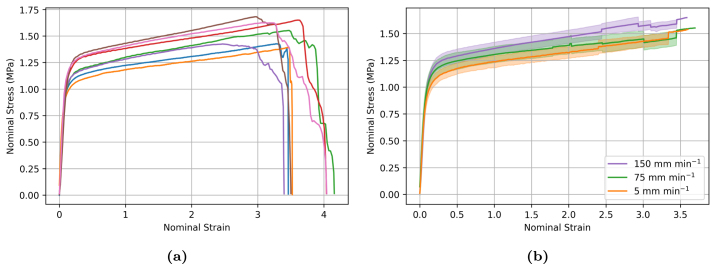
(a) Illustration of experimental variability in the stress–strain response of electrospun filaments tested at 75 mm min^−1^ using screw-side grips. (b) Averaged uniaxial response of filaments tested at extension rates of 5, 75, and 150 mm min^−1^.

**Fig. 12:**
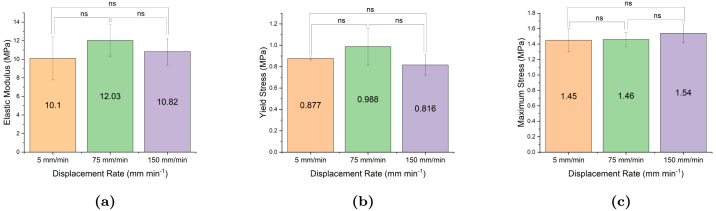
Summary statistics on the effect of strain rate on the (a) elastic modulus, (b) yield stress ($\sigma_{y}$), and (c) maximum stress ($\sigma_{max}$). Error bars represent standard deviation. ns = not significant, *p<0.05, **p<0.01, ***p<0.001, ****p<0.0001.

Bollard grips are a commonly utilised for rope testing because they diffuse stress concentration from the clamps by distributing the pulling tension over a larger surface area, enabling samples to be stretched to larger strains. However, the results shown in Fig. 9(a) suggest that they are prone to significant experimental variability. Therefore, we considered screw-side grips as an alternative method to bollard grips, and investigated whether they could provide more accurate measurements. Fig. 11(a) illustrates the experimental variability in the measured stress–strain curve for samples tested at 75 mm min^−1^. Similar to the bollard grips, filaments tested using screw-side grips still exhibit a large variability in the stress–strain response. Different from the response obtained using bollard grips, the stress–strain response is now decomposed into a linear regime followed by a plastic regime with moderate linear hardening, without re-hardening at large strains. Filaments are unable to reach the same stresses as bollard grips and fail around strains of about four, which is approximately three times less than the strain obtained with bollard grips. The average response of filaments tested using screw-side grips at different displacement rates is shown in Fig. 11(b), with the shadowed regions showing the standard deviation at each strain value. In this case, the average curves at different rates follow the expected trend in the early loading stage, with the slower loading rate exhibiting the smaller yield stress. We note however that the averaged curve for 75 mm min^−1^ went below the averaged curve for 5 mm min^−1^ at larger strains, which could be due to damage phenomena. Statistical analysis results shown in Fig. 12 reveal that the observed difference in stress values between different extension rates are not statistically significant.

### 3.5 SEM characterisation

#### SEM of pre-stretched filaments (bollard grips).

**Fig. 13:**
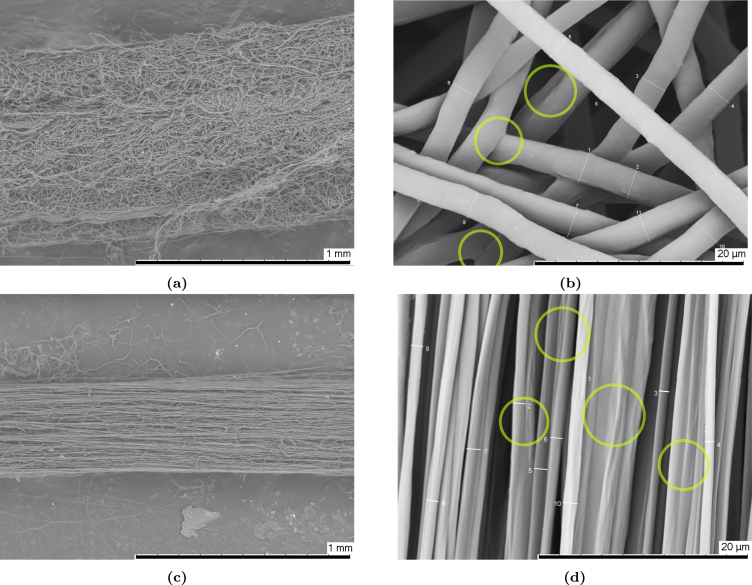
Micrographs showing the surface topology of (a)–(b) un-stretched and (c)–(d) stretched (${ɛ}_{n}=9$) electrospun filaments. Yellow circles in figures (b)–(d) highlight fibre fusion.

The morphology of ES filaments in the as-produced and stretched states are shown in Fig. 13 for two levels of magnification. During the manufacturing, ES fibres are deposited in a random fashion onto the fine metallic wire. The fibres conglomerate to produce filaments with an isotropic, porous, and tightly packed network structure, shown in Fig. 13(a). As-produced filaments are free from beads and other imperfections. However, fibre fusion is observed, as highlighted in Fig. 13(b). Fig. 13(c) shows the micrograph of a filament stretched by ten times its original length. Comparing Fig. 13, Fig. 13, a significant reduction in filament diameter is observed due to fibre stretching, as well as fibre alignment in the stretching direction. The reduction in overall filament diameter causes fibres to come into contact with each other, leading to fibre fusion and coalescence, shown in Fig. 13(d). The diameter of the fibres themselves is also reduced. Although not visible from the SEM micrographs, un-stretched and stretched filaments exhibit different optical properties. Un-stretched filaments were opaque white and flexible, whilst stretched filaments displayed shiny translucent optical properties, which can be attributed to strain-induced crystallisation.

**Fig. 14:**
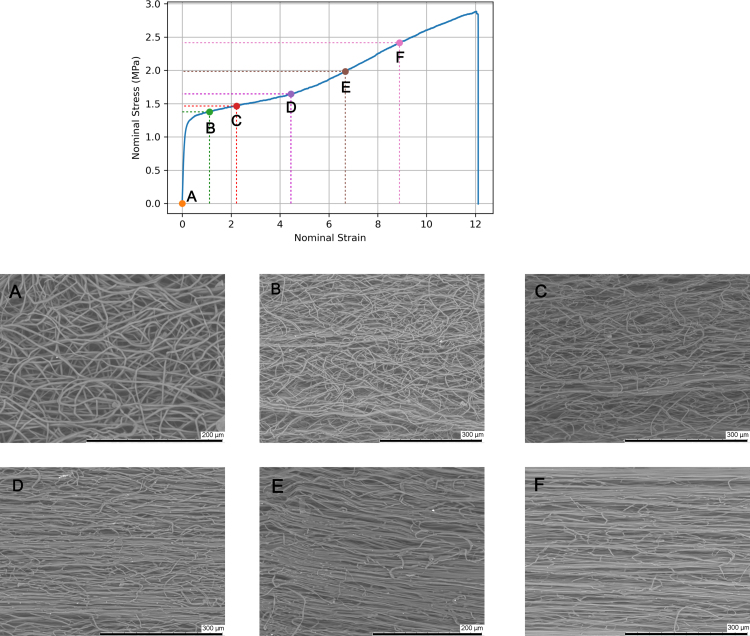
SEM micrographs showing the microstructural evolution of filaments stretched with bollard grips.

We further evaluated the fibre morphology progression at different stages of the stress–strain to better comprehend the key deformation mechanisms influencing the mechanical properties of the material. SEM micrographs corresponding to filaments stretched to different strains are shown in Fig. 14, alongside the typical stress–strain response. In the as-produced state (Fig. 14(A)), fibres are randomly oriented with a homogeneous diameter size and the topology contains large pores. Upon applying tension and reaching strains of one or two (Fig. 14(B) and (C)), the stress–strain curve indicates that the material is plastically deforming. Micrographs reveal that a small numbers fibres are commencing to align in the loading direction, while the microstructure remains mainly isotropic. Comparing micrographs Fig. 14(B) and (C), there are more aligned fibres at stage (C) compared to (B). Upon reaching stage (D), the fibre morphology has completely altered with the majority of fibres are aligned in the loading direction, the topological porosity is minimal and fibres appear to coalesce. At stage (E), the fibres remain aligned in the loading direction, but appear to coalesce further. Finally, reaching stage (F), the fibre morphology remains the same, but the fibre diameters reduced relative to stage (E).

The porosity, filament diameter, and fibre diameter were measured for every strain and the results are summarised in Table 3. Analysing differences between the initial state (${ɛ}_{n}=0$) and the final recorded strain (${ɛ}_{n}=9$), the porosity decreased by 42.7%, the filament diameter by 46.4%, and the fibre diameter by 62.5%. We also calculated the overall volume change of the filament J using the following formula:

(4) $$J=\frac{Ld^{2}}{L_{0}d_{0}^{2}}=\lambda \frac{d^{2}}{d_{0}^{2}}$$

where $d_{0}$ and d respectively refer to the initial and current diameter of the filament. Values are reported in Table 3. Calculated values show that the filament undergoes significant volume expansion during stretching, with J>1 despite the reduction in filament diameter during stretching, due to the very large axial stretch. We also estimated the axial stretch of the fibres assuming that they deform under constant volume. The fibre stretch $\lambda_{f}$ was calculated as:

(5) $$\lambda_{f}=\frac{d_{f0}^{2}}{d_{f}^{2}}$$

where $d_{f0}$ and $d_{f}$ respectively refer to the initial and current diameter of the fibres. Fibre stretch values are also reported in Table 3. Comparing the fibre stretch $\lambda_{f}$ to the filament stretch $\lambda ={ɛ}_{n}+1$, it can be observed that fibres deform on average less than the filament as a whole. This indicates that the deformation of the filament is not solely accommodated by the stretching of the fibres, and that other mechanisms are involved as well, such as the untangling and re-alignment of the fibres. The stretching of fibres is particularly prominent in the re-hardening region, where the majority of fibres are aligned in the loading direction: between (B) and (D), the fibre stretch increases by 95%, whereas it increases by 178% between (D) and (F).

An additional important observation is the large standard deviation in measured fibre diameters between stages (A) and (D), indicating that a proportion fibres in the loading direction may have been stretched already, whilst others are only beginning to stretch as they are recruited in the loading direction. The standard deviation is reduced in the re-hardening regions (i.e. (E) and (F)), as fibres are gradually approaching the same value. Once fibres reach their maximum extensibility, filament failure occurs.

#### SEM of pre-stretched filaments (screw-side grips).

**Fig. 15:**
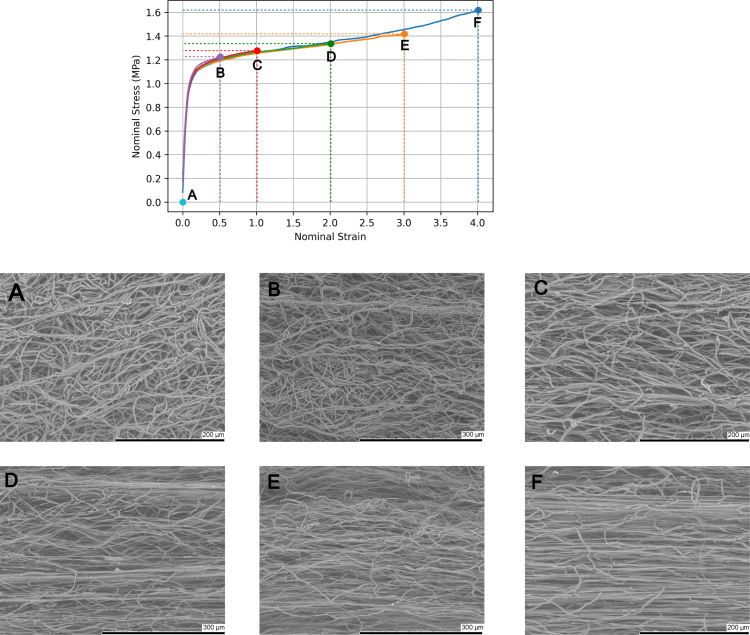
SEM micrographs showing the microstructural evolution of filaments with screw-side grips.

SEM micrographs corresponding to filaments stretched to different strains using screw-side grips are shown in Fig. 15. The results are similar to corresponding results obtained by pre-stretching the filaments using bollard grips. For un-stretched filaments, fibres initially have uniform diameter and are randomly oriented (Fig. 15(B)). Upon the application of the load, fibres begin to untangle and align in the loading direction, as shown in Fig. 15(B and C). At a strain of around 2, the filament texture is clearly anisotropic, with many fibres aligned in the direction of loading (Fig. 15(D) and (E)). However, there are still undulating fibres that are aligned in the loading direction but are tortuous and not fully stretched. Upon reaching a strain of around four (Fig. 15(F)), most tortuous fibres have engaged with the applied tension. Subsequently, fibres contract onto a tightly packed structure, thus reducing the filament diameter. The porosity, filament, and fibre diameters were measured for every strain and the results are summarised in Table 4. Results show that surface porosity reduces from 25% ± 1 to 16% ± 1 and the filament diameter reduces by 35.8% between strains of 0 and 4. Furthermore, fibres also reduced in diameter by 54.3%. The filament volume ratio J was computed using Eq. (4), and the fibre stretch was computed using Eq. (5). Calculated values also reported in Table 4.

**Table 3:** The surface porosity ϕ, filament diameter, filament volume ratio J, fibre diameter and fibre stretch $\lambda_{f}$ for samples stretched to various strains ${ɛ}_{n}=\lambda -1$ using bollard grips.

λ	Stage	ϕ (%)	Filament diameter (μm)	J	Fibre diameter (μm)	$\lambda_{f}$
1	A	24.19	832.2 ± 10.1	1	3.2 ± 0.5	1
2	B	21.01	761.6 ± 12.3	1.68	2.8 ± 0.7	1.31
3	C	19.51	679.1 ± 14.0	2.00	2.3 ± 0.8	1.94
5	D	17.40	624.7 ± 4.5	2.82	2.0 ± 0.8	2.56
8	E	15.48	574.6 ± 16.4	3.81	1.5 ± 0.3	4.55
10	F	13.85	446.1 ± 2.9	2.87	1.2 ± 0.3	7.11

#### In-situ SEM of stretched filaments.

**Fig. 16:**
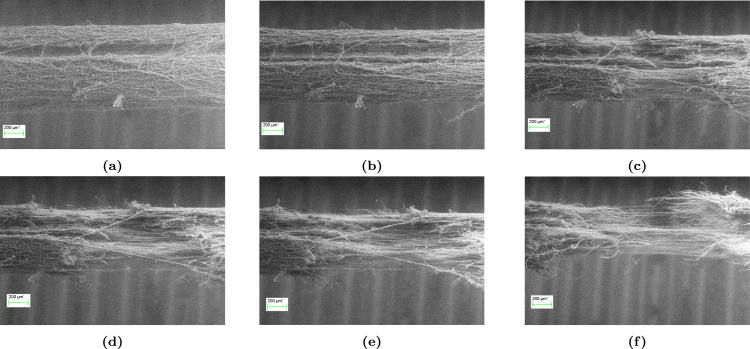
*In-situ* SEM micrographs showing the continuous microstructure evolution of the electrospun filaments stretched with screw-side grips. The fracture process involves the rupture of fibres from the outer layer inwards.

*In-situ* SEM was also conducted to investigate microstructural elements that cannot be captured through the previously used SEM method, such as filament failure. Fig. 16 shows a series of SEM micrographs which exhibit the sequence of events to filament failure. The imprint of the collecting wire is also visible on these images. In the initial state (Fig. 16(a)) filaments are randomly oriented and gradually begin to align in the loading direction upon the application of load alongside contracting in the transverse direction to loading (Fig. 16(b)). Subsequently, fibres in the outer layer rupture (Fig. 16(c)), creating a neck where deformation localises and where fibres are more aligned relative to the rest of the filament. Fibre failure progressively increases, rupturing fibres from the outer layer towards the centre, as shown in Figs. 16(d)–16(f).

**Table 4:** The surface porosity ϕ, filament diameter, filament volume ratio J, fibre diameter and fibre stretch $\lambda_{f}$ for samples stretched to various strains ${ɛ}_{n}=\lambda -1$ using screw-side grips.

λ	Stage	ϕ (%)	Filament diameter (μm)	J	Fibre diameter (μm)	$\lambda_{f}$
1	A	25.34	859.9 ± 23.7	1	3.5 ± 0.7	1
1.5	B	22.07	815.7 ± 19.8	1.35	3.1 ± 0.6	1.27
2	C	21.53	737.5 ± 15.1	1.47	2.7 ± 0.7	1.68
3	D	18.15	611.9 ± 14.9	1.52	2.3 ± 0.7	2.32
4	E	17.46	587.1 ± 13.3	1.86	1.7 ± 0.5	4.24
5	F	16.89	552.2 ± 8.9	2.06	1.6 ± 0.5	4.79

## 4 Phenomenological model

### 4.1 Model description

**Fig. 17:**
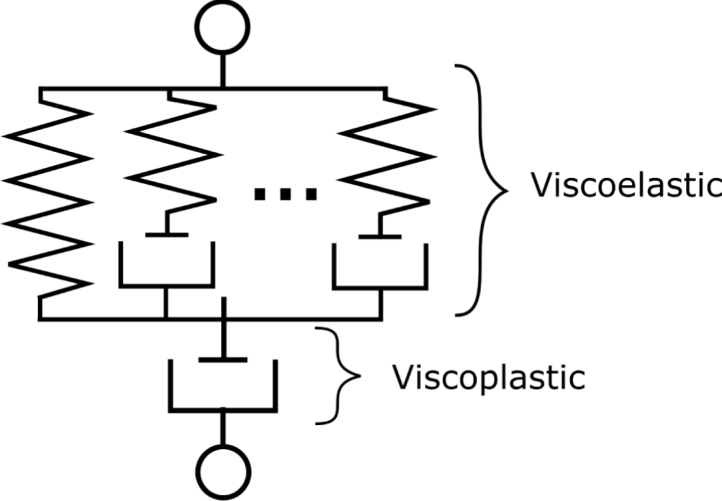
1D representation of the constitutive model, consisting of generalised Maxwell model in series with a viscoplastic dashpot.

We developed a rate-dependent constitutive model to capture the filament response up to large deformations. In the proposed model, the stress response is decomposed into viscoelastic and viscoplastic components. The viscoelastic component aims to describe intermolecular interactions within the fibres, whereas the viscoplastic component aims to capture the inter-fibre interactions and reorganisation of the fibre network during stretching, as well as the re-hardening at large deformations, attributed to the stretching of the individual fibres. A 1D rheological depiction of the model is shown in Fig. 17. The framework consists of a generalised Maxwell model in series with a J${}_{2}$ viscoplastic dashpot. This model was adopted as the simplest possible combination of two standard constitutive models respectively used to represent linear viscoelastic behaviour and elastic-viscoplastic behaviour. The model is versatile, allowing an arbitrary number of relaxation times in the viscoelastic model, as well as general forms of viscoplastic and hardening functions. Furthermore, an efficient algorithm for the numerical implementation of this model was developed by Miled et al. (2011) and is adopted here.

We limit the presentation of the model to the one-dimensional case, given the geometry of the filament and the uniaxial tension loading condition. Let λ be the tensile stretch. The logarithmic (true) strain is given by:

(6) $$ɛ=\ln\left(\lambda \right)$$

The true strain is additively decomposed into viscoelastic and viscoplastic components:

(7) $$ɛ={ɛ}^{ve}+{ɛ}^{vp}$$

According to the Boltzmann hereditary integral (Bergstrom, 2015, Brinson, 2015), the stress is related to the viscoelastic strain by:

(8) $$\sigma \left(t\right)=\int_{0}^{t}E\left(t-\tau \right)\frac{\partial {ɛ}^{ve}}{\partial \tau }d\tau$$

where E(t) is the viscoelastic modulus of the generalised Maxwell model, which can be expressed using Prony series as:

(9) $$E\left(t\right)=E_{\infty }+\overset{N}{\underset{k=1}{\sum }}E^{\left(k\right)}\exp\left(-\frac{t}{\tau^{\left(k\right)}}\right)$$

where $E_{\infty }$ is the long-term modulus, $\tau^{\left(k\right)}$ are the viscoelastic relaxation times and $E^{\left(k\right)}$ the corresponding moduli of the N viscoelastic branches in the generalised Maxwell model. Substituting expression (9) into the hereditary integral (8), we obtain the stress as:

(10) $$\sigma \left(t\right)=E_{\infty }{ɛ}^{ve}+\overset{N}{\underset{k=1}{\sum }}\sigma^{k}\left(t\right)$$

where

(11) $$\sigma^{k}\left(t\right)=E^{\left(k\right)}\exp\left(-\frac{t}{\tau^{\left(k\right)}}\right)\int_{-\infty }^{t}\exp\left(\frac{\tau }{\tau^{\left(k\right)}}\right)\frac{\partial {ɛ}^{ve}}{\partial \tau }d\tau$$

The viscoplastic strain is described using standard $J_{2}$ viscoplasticity with isotropic hardening. The yield function is given by:

(12) $$f\left(\sigma_{eq},p\right)=\sigma_{eq}-\left(\sigma_{y}+R\left(p\right)\right)$$

where $\sigma_{eq}=\left|\sigma \right|$ is the equivalent stress (in 1D), $\sigma_{y}$ is the yield stress, and R(p) is the hardening function, which depends on the accumulated plastic strain p. Note that the stress is always positive in the loading scenarios considered in the present paper. For uniaxial tension, the accumulated plastic strain is given by:

(13) $$p\left(t\right)=\int_{0}^{t}\overset{˙}{p}\left(\tau \right)d\tau \;\text{with}\;\overset{˙}{p}=\left|{\overset{˙}{ɛ}}^{vp}\right|$$

The uniaxial flow rule for the viscoplastic strain is simply expressed as:

(14) $${\overset{˙}{ɛ}}^{vp}=\overset{˙}{p}\text{sign}\left(\sigma \right)$$

and the plastic multiplier is defined by a viscoplastic function $g_{v}$:

(15) $$\left\{\begin{array}{cc} \overset{˙}{p}=0,\; & \;\text{if}f\leq 0\text{(viscoelasticity)}\\ \overset{˙}{p}=g_{v}\left(\sigma_{eq},p\right)>0,\; & \;\text{if}f>0\text{(viscoelasticity + viscoplasticity)} \end{array}\right)$$

To capture rehardening at large deformation, we take the hardening function of an exponential form:

(16) $$R\left(p\right)=k\exp\left(np\right)$$

where k is the hardening coefficient and n the hardening exponent. The viscoplastic function is defined by Norton’s power law:

(17) $$g_{v}\left(\sigma_{eq},p\right)=\left\{\begin{array}{cc} \frac{\sigma_{y}}{\eta }{\left(\frac{f}{\sigma_{y}}\right)}^{m},\; & \;\text{if}f>0\\ 0,\; & \;\text{otherwise} \end{array}\right)$$

where η and m are the viscoplastic modulus and exponent, respectively.

The numerical implementation of the viscoelastic–viscoplastic model is based on the fully-implicit algorithm proposed by Miled et al. (2011). In brief, the algorithm involves a viscoelastic predictor step followed by a viscoplastic correction to satisfy the yield condition. The viscoplastic correction step is identical to the classical elasto-viscoplastic return mapping algorithm, which is achieved via the introduction of incremental relaxation moduli. Another advantage of the method is that it does not require the storage of the entire deformation history, but only the state variables at the previous times step. We refer to the paper by Miled et al. (2011) for details about the time-discretisation algorithm.

### 4.2 Comparison between model predictions and experimental data

**Fig. 18:**
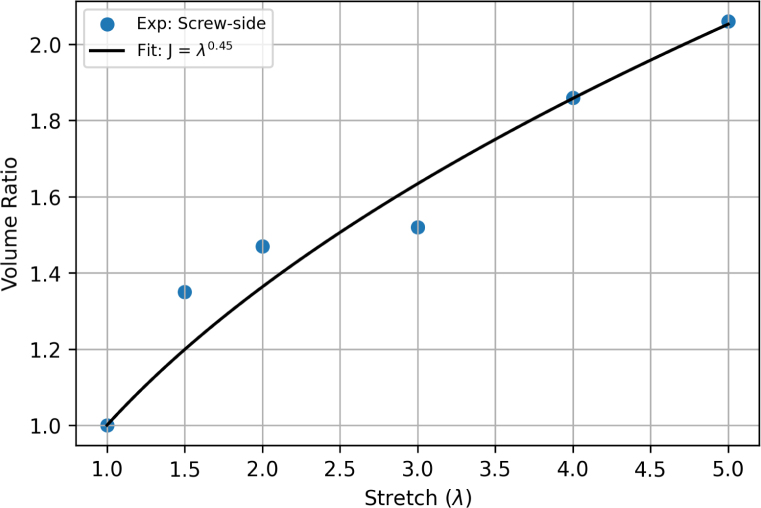
Relationship between stretch and volume ratio. The solid black line is the empirical fitting function $J=\lambda^{0.45}$.

We have calibrated the model based on experimental data obtained using the screw-side grips. Experimental and model results are both presented using true stress and strain. In the experiments, the true strain was calculated from the nominal strain ${ɛ}_{n}$ using the relation $ɛ=\ln\left(1+{ɛ}_{n}\right)$, whereas the true stress was calculated from the nominal stress $\sigma_{n}$ as $\sigma =\sigma_{n}\frac{\lambda }{J}$. Here, the volume change J was determined as a function of the stretch by fitting the values reported in Table 4 with the empirical formula:

(18) $$J=\lambda^{0.45}$$

which satisfies the requirement that J(1)=1. The empirical fitting of the volume ratio as a function of applied stretch is shown in Fig. 18.

Model parameters were identified in the following way. The strain rate used in the model was the same as in the experiments. As an initial guess, the instantaneous apparent modulus ($E_{0}=E_{\infty }+\sum E_{i}$) and yield stress were initially calibrated based on the experimentally measured apparent elastic modulus and yield stress, respectively. For simplicity, we considered N=2 Maxwell elements in the viscoelastic model, which we found sufficient to obtain good agreement with experimental stress–strain curves for the considered strain rates. The remaining viscoelastic and viscoplastic parameters were optimised using least square minimisation using Python scipy optimisation algorithm. After completing the optimisation, the apparent modulus was further adjusted to better capture the elastic unloading and reloading stages during non-monotonic loading, as shown below. The final parameters used to fit all the stress–strain curves are reported in Table 5.

We also attempted to fit the experimental stress–strain curves using the viscoelastic parameters identified from DMA measurements and reported in Table A.6. However, the model with viscoelastic parameters directly taken from DMA was not able to replicate the cyclic or stress relaxation behaviour, despite the much larger number of Maxwell elements (N=37). This is attributed to nonlinear viscoelastic effects, so that linear viscoelastic parameters identified under small strain loading conditions do not provide good predictions under large strains. In contrast, the viscoelastic model with N=2 and fitted parameters provides satisfying predictions but should be viewed as a heuristic approach to describe the nonlinear viscoelastic response for a limited range of strain rates. However, we have verified that the storage and loss modulus values predicted using the model with N=2 and parameters reported in Table 5 are reasonable in view of the experimental data reported in Fig. 6. Specifically, the nominal strain rate in the tensile test was converted to an equivalent frequency as ${\overset{˙}{ɛ}}_{n}\approx 4{ɛ}_{0}f$ (Song et al., 2023). For an applied displacement rate of 75 mm min^−1^, corresponding to ${\overset{˙}{ɛ}}_{n}=0.125$ s^−1^, the equivalent frequency is f=31.25Hz. According to the experimental master curves shown Fig. 6, the storage modulus at that frequency is 19.5 MPa, and the loss modulus is in the range 0.1–0.9 MPa. In comparison, the storage and loss moduli calculated from Eqs. (2), (3) using parameters in Table 5 and using ω=2πf are 25 MPa and 0.022 MPa, respectively, which is in broad agreement with the experimental data. Similar agreement was found for the two other loading rates.

**Fig. 19:**
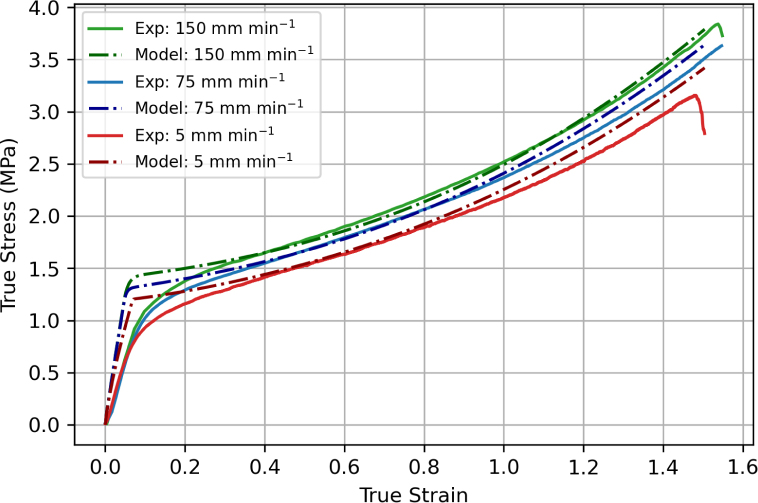
Comparison between experimental results and model predictions for the uniaxial tension response of filaments at different extension rates.

Model predictions are compared to experimental data for uniaxial tension at various displacement rates in Fig. 19. Consistent with the experimental observations, the model predicts an (apparent) linear elastic response, followed by plastic yielding and hardening. The slope of the initial elastic response is overestimated by the model, which was found necessary to capture the elastic response during the unloading and reloading stages in non-monotonic loading, as shown below.

**Table 5:** Model parameters used to reproduce the stress–strain response measured using screw-side grips.

Viscoelastic parameters		
Elastic moduli	$E_{\infty }$= 10 MPa	
	$E^{\left(1\right)}$= 8 MPa	$E^{\left(2\right)}$= 7 MPa
Relaxation times	$\tau^{\left(1\right)}$= 10.00 s	$\tau^{\left(2\right)}$= 2.00 s

Viscoplastic parameters		

Yield stress	$\sigma_{y}$= 0.7 MPa	
Hardening function	k= 0.5 MPa	n= 1.45
Viscoplastic function	η= 1 MPa s	m= 1

**Fig. 20:**
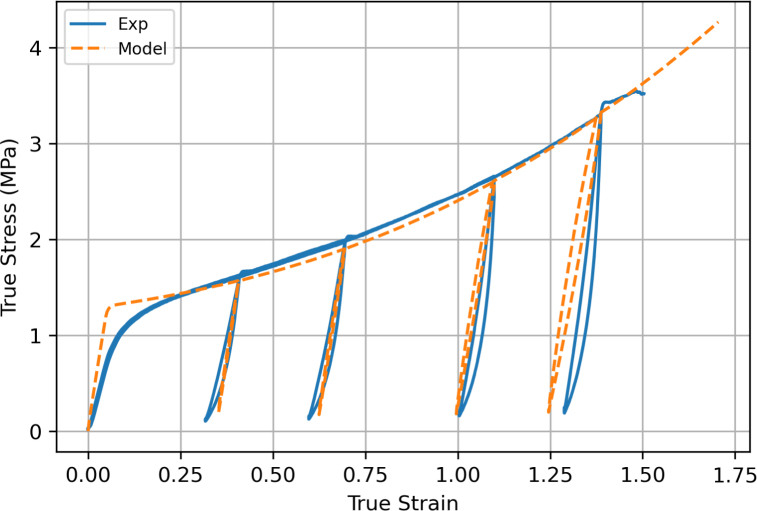
Comparison between experimental results and model predictions for the loading/unloading response of filaments at a displacement rate of 75 mm min^−1^.

The loading/unloading response using screw-side grips is shown in Fig. 20 for a displacement rate of 75 mm min^−1^. The model well predicts the unloading–reloading response at 0.4 and 0.7 strain, with a good prediction of the modulus and hysteresis loop. However, at larger strains of 1.1 and 1.4, the model predictions are less accurate. Nevertheless, the model continues to capture the progressive increase in hysteresis and the overall material response with good agreement.

**Fig. 21:**
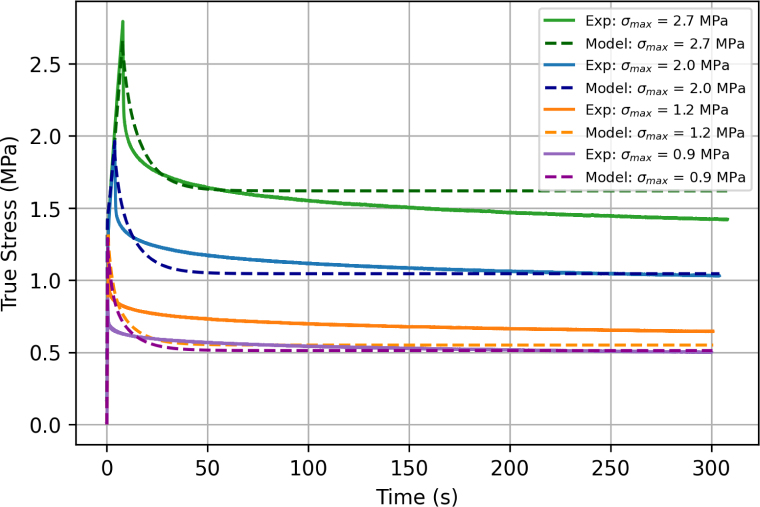
Comparison between experimental results and model predictions for the stress relaxation response of filaments loaded to a given maximum stress value $\sigma_{max}$ at a displacement rate of 75 mm min^−1^, and held at constant elongation afterwards.

Model predictions in relaxation tests are shown in Fig. 21, along with experimental data. Different curves correspond to different values of the maximum (true) stress reached during the initial loading stage at 75 mm min^−1^. The displacement was then held constant while the stress relaxed. The model well captures the experimental response. In particular, the model is able to predict both the short-term and long-term responses for different values of the applied displacement.

## 5 Discussion

### 5.1 Mechanical behaviour

Based on the typical experimental stress–strain curve and the SEM micrographs taken at various deformation stages shown in Fig. 14, we decompose and interpret the mechanical response of filaments in four stages. The first stage is the linear (visco)elastic response of the filament, which occurs up to a nominal strain of about 0.08±0.01. During the linear deformation stage, the filament consists of a network of fibres held together by physical and chemical cross-link points. Fibres act as the load bearing components, whereas the fibre junctions as the load-transferring elements (Rawal, 2022). Physical cross-links consist in entanglements and friction points between individual fibres. Chemical cross-links here refer to fused contact points, shown in Fig. 13(b), resulting from the forceful contact between wet fibres during deposition and increasing the interconnection of the network (Bhardwaj and Kundu, 2010, Richard-Lacroix and Pellerin, 2013, Suwantong, 2016). In an elastic network of fibres, known factors influencing the elastic modulus include the porosity, the elastic modulus of the fibres, the fibre diameter and curvature, and the density of both physical and chemical cross-links (Pai et al., 2011). For example, in a previous study on polyamide-6 mats under tensile loading, Molnar et al. (2012) found that fibre bonding and friction play an important role in maintaining the structural integrity of the network.

The second stage involves the plastic yielding of the filament, which is attributed to the gradual breaking of the cross-links. The large variability in yield point (Fig. 9(a)) could be related to the variation in density and strength of the cross-link points from sample to sample, as well as to pre-stress introduced when tightening the specimen onto the grips, which could rupture some of the cross-links. During yielding, the overall deformation of the filament is primarily accommodated by progressive fibre alignment in the loading direction, hence the macroscopic stress shows only small hardening. Additional mechanisms that may play a role are layer slippage, considering electrospinning is a layer-by-layer deposition technique, there may be layer slippage concurrently occurring with the re-alignment of fibres.

The third stage involves re-hardening, where the stress–strain gradient steepens at large deformations. We attribute re-hardening to the stretching of individual fibres aligned in the loading direction (Fig. 14). The large stretching of individual fibres is evidenced by the reduction in diameter (by 40% between stages (D) and (F)), which implies stretching of the fibres assuming near incompressibility of the polymer. As the fibres are stretched to large deformations, they themselves undergo re-hardening, which is primarily attributed to the limit extensibility of stretched polymer chains in the PCL network. Strain-induced crystallisation probably plays a minor role in the overall rehardening, given the small increase in crystallinity (between stages (E) and (F) the recorded crystallinity value increased only by 3%, cf. Table 1). Several groups have studied the evolution of the mechanical properties of fibres in relation to their diameter. For example, Tan et al. (2004) found that a reduction in fibre diameter resulted in an increase in Young’s modulus of PLLA nanofibres. Zussman et al. (2006) showed that ES fibres made of Nylon-6,6 possess a skin-core morphology. The skin consists of oriented layered planes with crystallites, but are misoriented with respect to the core fibre axis. As the fibre diameter is reduced, a critical diameter is reached where the skin and core are similarly oriented, correlating with an increase in molecular orientation. Such results have been further supported by experimental tests conducted by Lim et al. (2008) and Arinstein et al. (2007), which showed that the Young’s modulus, molecular orientation, and crystallinity increased with smaller fibre diameters due to the greater restriction of polymer conformation.

The final stage is filament failure, corresponding to the sudden stress drop in the stress–strain response (Fig. 9). SEM micrographs in Fig. 16 reveal that filament rupture occurs in localised regions where fibres are highly aligned in the loading direction and have reached their extensibility limit. It is possible that cross-section inhomogeneities induced by the detachment of the filament from the collecting wire also impact filament failure. However, micrographs do not indicate any localisation of damage near the wire imprint. We hypothesise that failure occurs through a compensation mechanism, whereby failure of an individual fibre involves load redistribution over the remaining fibres. The process is repeated as the macroscopic load in further increased, until catastrophic failure. Similar findings have been reported by Farukh et al., 2014a, Farukh et al., 2014b, who investigated the progressive damage of thermally bonded non-woven networks. These studies reported that network failure often occurred at advanced stages of stretching when fibres have re-oriented and straightened in the loading direction. Fibre failure occurs once their stress or strain threshold was reached. Progressive failure of the fibres would gradually lead to the overall rupture of the network. Fibres can also fail due to the gradual growth of damage through other mechanisms. As an example, Ridruejo et al. (2011) reported that damage occurs at the very early stages of deformation. Micro-damage in fibres is initially triggered by chemical bond breakage formed at wet contact points and further develops due to frictional slippage between fibres during plastic deformation. This has phenomena has been confirmed by Chidambaram et al. (2000) and Farukh et al. (2014a), who argue that bonding process changes the molecular orientation of fibres in the chemical bond periphery, leading to a decrease in elastic modulus and strength of the fibres.

Our experimental results show that the mechanical response of ES PCL filaments is rate dependent. This is most clearly seen from the stress relaxation tests, Fig. 8, as well as from the presence of hysteresis during loading/unloading sequences. A strain rate effect is also observed on the yield stress (Fig. 9, Fig. 11), although definitive conclusions are made difficult by the experimental variability. Predictions of our phenomenological model confirm that the material response is viscoelastic–viscoplastic, and that both components need to be taken into account to capture all features of the material response, namely rate-dependent yield stress, hysteresis and stress relaxation. Rate-dependent behaviour of ES PCL has also been reported in the literature in the context of ES mats (Duling et al., 2008, Baker et al., 2015). Several mechanisms can influence the rate-dependent behaviour of filaments at both the fibre and polymer chain scale. At fibre level, mechanisms include the debonding of chemical and physical cross-link points, disentanglement, and slippage of fibres onto each other. The intrinsic response of the PCL chains constituting the fibres is also rate-dependent. The precise interplay between these mechanisms is still ambiguous and require further investigation.

The impact of moisture and wet conditions is an important factor to consider when evaluating the performance of electrospun (ES) filaments. The mechanical properties of ES filaments can vary significantly depending on the environment in which they are used. In this study, PCL ES filaments were produced in a controlled environment and stored in a desiccator prior to testing. However, all mechanical tests were conducted in environmental humidity conditions. In a recent work, Alharbi and Guthold (2024) compared the mechanical properties of hydrated and dry ES PCL nanofibers. Results showed that hydrated samples stretched slightly more and failed at earlier stresses, however, the differences were small. Further investigation is needed to fully understand the effects of hydration on ES filaments.

### 5.2 Reliability of mechanical testing

ES filaments were characterised utilising two types of mechanical grips, namely screw-side grips and bollard grips, in order to evaluate the impact of the testing method on the measured properties of filaments prepared in the exact same conditions. Indeed, difficulties in comparing experimental measurements for non-woven textiles with similar preparation conditions but tested following different protocols were recently highlighted by Rashid et al. (2021). Our results show that measured properties are strongly impacted by the type of grips. In particular, filaments tested using screw-side grips failed at much smaller strains, which prevented us to observe the re-hardening behaviour at large deformation with this method. We hypothesise that gluing filaments to the cardboard (Fig. 2) locally prevents the untangling and alignment of the fibres during stretching, leading to stress concentration within the fibres near the grips and hence to premature failure. In an attempt to alleviate the local pressure at the grips, square rubber sheets were inserted between the cardboard and the grip face, however, no difference was noted. Another difference is that specimens tested using screw-side grips (Fig. 11(a)) show the expected trend for PCL that testing at higher strain rate leads to higher stresses, e.g. Duling et al. (2008). However, statistical significance could not be established.

A key limitation associated with bollard grips tensile tests is that it is difficult to measure strain accurately. This could partially explain the large experimental variability and unexpected rate-dependency observed using these grips. First, the defined gauge length did not take into account the excess filament wrapped around the bollard, which can also be stretched and contribute to the prescribed cross-head displacement. Second, the axial strain was not homogeneous within the gauge region with some regions showing delayed activation. Strain delocalisation is attributed to hardening (possibly assisted by strain-induced recrystallisation) in the highly stretched regions, preventing necking. As a result, reported measures of strains should be seen as average values. To address this, we have attempted to measure the local axial strain using digital image correlation (DIC). However, we encountered several obstacles. First, creating a micron- to nano-sized speckle pattern on the porous structure proved difficult, as the paint seeped into the pores. Second, our available equipment (a Point Grey camera paired with a Nikkor 105 mm lens) did not offer sufficient magnification to capture the nano-sized speckle pattern clearly. Third, due to the high strain experienced by the filaments, the tracking pattern often moved out of the camera’s field of view. For these reasons, strain characterisation using image-based methods was not included in the scope of this study, and further investigations are required.

### 5.3 Implications for biomedical application

Continuous ES filaments as studied in this work offer great promises as tissue engineering scaffolds for tendon and ligament repair, thanks to their porous architecture and ability to be braided into yarns with customisable mechanical properties (Abhari et al., 2018a, Abhari et al., 2018b). The natural anterior cruciate ligament bears an average daily load in the range 0–300 N, with rupture typically occurring at forces around 2300 N in healthy adults (Marieswaran et al., 2018). While a single ES filament has an average maximum tensile force of the order of 1.5 N (based on the tensile response shown in Fig. 7), previous work by Mouthuy and coworkers (Mouthuy et al., 2015, Savić et al., 2021) has shown that the strength of yarns increases almost linearly with the number of filaments. In particular, Savić et al. (2021) showed that PCL yarns can achieve ultimate tensile forces up to 270 N.

**Fig. 22:**
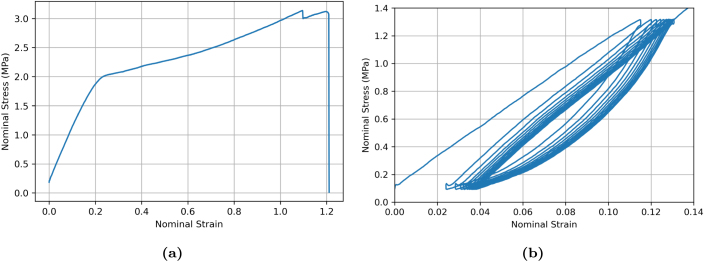
Representative nominal stress–strain response of a filament pre-stretched by a factor of eight during (a) monotonic loading and (b) cyclic loading up to a nominal stress of 1.3 MPa. All mechanical tests were conducted at 75 mm min^−1^ using bollard grips. Nominal strains reported in the figure were calculated using the pre-stretched state as the reference state.

Mouthuy et al. (2015) also highlighted the benefit of pre-stretching the filaments prior to further assembly into braided structures. Pre-stretching the filaments increases their apparent stiffness and yield strength while reducing the ability of the material to deform plastically, which could compromise material performance. Furthermore, stretched filaments possess a highly aligned microfibrous structure, closely mimicking the extracellular matrix (ECM) of natural ligaments, as also demonstrated in the present work, see Fig. 14. Fig. 22a shows the representative response of a filament that has been previously pre-stretched by a factor of eight using bollard grips, following the procedure described in Section 2.4. The apparent modulus of the pre-stretched filament is 9.5 MPa, which is slightly higher than the un-stretched average value of 9.1 MPa reported in Section 3.3. However, the yield stress raised to 1.8 MPa which is twice the yield stress of the un-stretched samples, also reported in Section 3.3. Fig. 22b shows the typical response of a pre-stretched filament during 10 loading/unloading cycles with the maximum force set to 0.7 N, corresponding to a nominal stress of 1.3 MPa. While the specimen still exhibits some residual plastic deformation after the first unloading, the plastic deformation did not increase significantly in subsequent cycles and the hysteresis loop stabilised to a steady shape. In other words, pre-stretched filaments exhibit nearly viscoelastic behaviour in a strain range 0%–12%. This apparent viscoelastic behaviour is attributed to the disentanglement and reorientation of the fibres, which was completed during the pre-stretching stage, exhausting the capability of further plastic deformation in the pre-stretched specimen.

Fatigue response of ES filaments is also critical for tendon and ligament repair applications. While a complete fatigue characterisation of the ES filaments is beyond the scope of thie work, we did conduct some preliminary tests on pre-stretched filaments. Specifically, pre-stretched filaments were tested using the DMA instrument at 1 and 5 Hz at 1% strain for 18 h. For each tested frequency, the filament was able to withstand 324,000 cycles and exhibited minimal changes in dimension, storage and loss modulus over time. These preliminary results suggest that ES filaments are able to maintain mechanical stability for extended periods of time.

**Table A.6:** Moduli $E^{\left(i\right)}$ (MPa) and relaxation times $\tau^{\left(i\right)}$ (s) used to fit the experimental master curves in Fig. 6 using the Generalised Maxwell model. The relaxed modulus is $E_{\infty }=10\;\mathrm{MPa}$.

$E^{\left(1\right)}$ = 0.66	$\tau^{\left(1\right)}=3.2\times 10^{7}$	$E^{\left(14\right)}$ = 1.46	$\tau^{\left(14\right)}=3.2\times 10^{-6}$	$E^{\left(27\right)}$ = 5.78	$\tau^{\left(27\right)}=3.2\times 10^{-19}$
$E^{\left(2\right)}$ = 0.99	$\tau^{\left(2\right)}=3.2\times 10^{6}$	$E^{\left(15\right)}$ = 1.64	$\tau^{\left(15\right)}=3.2\times 10^{-7}$	$E^{\left(28\right)}$ = 4.91	$\tau^{\left(28\right)}=3.2\times 10^{-20}$
$E^{\left(3\right)}$ = 1.12	$\tau^{\left(3\right)}=3.2\times 10^{5}$	$E^{\left(16\right)}$ = 1.91	$\tau^{\left(16\right)}=3.2\times 10^{-8}$	$E^{\left(29\right)}$ = 3.90	$\tau^{\left(29\right)}=3.2\times 10^{-21}$
$E^{\left(4\right)}$ = 1.12	$\tau^{\left(4\right)}=3.2\times 10^{4}$	$E^{\left(17\right)}$ = 2.31	$\tau^{\left(17\right)}=3.2\times 10^{-9}$	$E^{\left(30\right)}$ = 2.90	$\tau^{\left(30\right)}=3.2\times 10^{-22}$
$E^{\left(5\right)}$ = 1.05	$\tau^{\left(5\right)}=3.2\times 10^{3}$	$E^{\left(18\right)}$ = 2.85	$\tau^{\left(18\right)}=3.2\times 10^{-10}$	$E^{\left(31\right)}$ = 2.05	$\tau^{\left(31\right)}=3.2\times 10^{-23}$
$E^{\left(6\right)}$ = 0.97	$\tau^{\left(6\right)}=3.2\times 10^{2}$	$E^{\left(19\right)}$ = 3.52	$\tau^{\left(19\right)}=3.2\times 10^{-11}$	$E^{\left(32\right)}$ = 1.44	$\tau^{\left(32\right)}=3.2\times 10^{-24}$
$E^{\left(7\right)}$ = 0.91	$\tau^{\left(7\right)}=3.2\times 10^{1}$	$E^{\left(20\right)}$ = 4.28	$\tau^{\left(20\right)}=3.2\times 10^{-12}$	$E^{\left(33\right)}$ = 1.14	$\tau^{\left(33\right)}=3.2\times 10^{-25}$
$E^{\left(8\right)}$ = 0.91	$\tau^{\left(8\right)}=3.2\times 10^{0}$	$E^{\left(21\right)}$ = 5.08	$\tau^{\left(21\right)}=3.2\times 10^{-13}$	$E^{\left(34\right)}$ = 1.12	$\tau^{\left(34\right)}=3.2\times 10^{-26}$
$E^{\left(9\right)}$ = 0.95	$\tau^{\left(9\right)}=3.2\times 10^{-1}$	$E^{\left(22\right)}$ = 5.82	$\tau^{\left(22\right)}=3.2\times 10^{-14}$	$E^{\left(35\right)}$ = 1.30	$\tau^{\left(35\right)}=3.2\times 10^{-27}$
$E^{\left(10\right)}$ = 1.03	$\tau^{\left(10\right)}=3.2\times 10^{-2}$	$E^{\left(23\right)}$ = 6.93	$\tau^{\left(23\right)}=3.2\times 10^{-15}$	$E^{\left(36\right)}$ = 1.55	$\tau^{\left(36\right)}=3.2\times 10^{-28}$
$E^{\left(11\right)}$ = 1.13	$\tau^{\left(11\right)}=3.2\times 10^{-3}$	$E^{\left(24\right)}$ = 6.71	$\tau^{\left(24\right)}=3.2\times 10^{-16}$	$E^{\left(37\right)}$ = 1.65	$\tau^{\left(37\right)}=3.2\times 10^{-29}$
$E^{\left(12\right)}$ = 1.23	$\tau^{\left(12\right)}=3.2\times 10^{-4}$	$E^{\left(25\right)}$ = 6.73	$\tau^{\left(25\right)}=3.2\times 10^{-17}$		
$E^{\left(13\right)}$ = 1.33	$\tau^{\left(13\right)}=3.2\times 10^{-5}$	$E^{\left(26\right)}$ = 6.41	$\tau^{\left(26\right)}=3.2\times 10^{-18}$		

## 6 Conclusion

Mechanical properties of novel electrospun poly-(ɛ-caprolactone) filaments were characterised and correlated with microstructural evolutions at the fibre network level. Thermal characterisation using DSC showed that the fibres are semi-crystalline, and that the degree of crystallinity slightly increases with the degree of pre-stretch. The small-strain viscoelastic behaviour of the filament was characterised by DMA, revealing a very broad transition region in the frequency domain for both the storage and loss moduli. The large deformation behaviour of the filament was characterised in tensile tests at various displacement rates. Results show that the filaments are viscoelastic–viscoplastic, involving rate-dependent plastic yielding followed by hardening at large deformations. The mechanical response is attributed to progressive fibre engagement and alignment, involving crosslink debonding and disentanglement, followed by stretching of individual fibres in the ES network as the deformation increases. These results support the idea of pre-stretching the filaments prior to their assembly into braided structures, in order to exhaust the plastic deformation capability of the filaments, effectively making them viscoelastic.

Our study highlights the importance of the testing method, by comparing responses measured using screw-side grips and bollard grips. While bollard grips enable the filaments to be stretched to larger strains and to exhibit significant re-hardening, they also cause inhomogeneous stretching of the filaments, which prevents accurate strain measurements. Variable degree of pre-stress introduced while mounting the specimens on the bollard grips might also contribute to the experimental variability.

A 1D phenomenological model was proposed to capture the viscoelastic-viscoplastic behaviour of electrospun filaments up to large strains. The model is able to capture the key features of the response, including the rate-dependent, loading/unloading response and stress relaxation with good agreement. However, the model is purely phenomenological. Future work includes the development of a micromechanical model, starting from a description of the individual fibres and their interaction in the non-woven network. This will provide further insight into the deformation behaviour of the filaments, which is critical to predict failure mechanisms in future medical textiles made of ES materials.

## CRediT authorship contribution statement

**Thales Zanetti Ferreira:** Writing – review & editing, Writing – original draft, Software, Methodology, Investigation, Formal analysis, Conceptualization. **Zhouzhou Pan:** Writing – review & editing, Software, Formal analysis. **Pierre-Alexis Mouthuy:** Writing – review & editing, Supervision, Resources, Methodology, Funding acquisition, Conceptualization. **Laurence Brassart:** Writing – review & editing, Writing – original draft, Supervision, Software, Resources, Methodology, Funding acquisition, Formal analysis, Conceptualization.

## Declaration of competing interest

The authors declare the following financial interests/personal relationships which may be considered as potential competing interests: Pierre-Alexis Mouthuy holds patent #WO20150403999A1 related to the filament fabrication technology. The other authors declare that they have no known competing financial interests or personal relationships that could have appeared to influence the work reported in this paper.

## Data Availability

Data will be made available on request.

## Appendix Fitting of DMA master curves

See Table A.6.
